# The Toxic Price of Beauty: Impact of Common Nail and Hair Care Products on Indoor Air Quality and Effectiveness of Air Purification

**DOI:** 10.7759/cureus.95720

**Published:** 2025-10-29

**Authors:** Jacqueline Grigoryants, Steve Miller

**Affiliations:** 1 Science Department, La Canada Preparatory School, La Canada, USA

**Keywords:** air purifier, beauty salons, indoor air quality, occupational exposure, particulate matter, volatile organic compounds

## Abstract

Introduction: Indoor air pollution caused by cosmetic products is an increasing public health concern, particularly for nail and hair salon workers frequently exposed to volatile organic compounds (VOCs) and fine particulate matter. These substances are associated with respiratory, neurological, and reproductive health risks.

Methods: Experiments were conducted in a sealed 270 ft³ room using a digital air quality monitor to measure the air quality index (AQI), total volatile organic compounds (TVOCs), formaldehyde (HCHO), and fine particulate matter (PM2.5). Nail polish, acetone-based remover, hairspray, and hair dye were individually and jointly tested. Two purification methods were compared: an exhaust fan (80 cubic feet per minute) and an air purifier equipped with HEPA and activated carbon filters (239 cubic feet per minute).

Results: All tested products worsened indoor air quality, with hairspray producing the greatest increase in pollutants. Combined product use resulted in an AQI exceeding 200 and PM2.5 above 200 µg/m³. The HEPA + activated carbon air purifier restored air quality to safe levels within one minute, while the exhaust fan produced only modest improvements over 15 minutes.

Conclusion: Common nail and hair care products significantly degrade indoor air quality. Air purifiers with HEPA and activated carbon filters are highly effective in removing harmful airborne compounds and should be routinely used in salons to protect workers and clients from long-term health risks.

## Introduction

Indoor air pollution from cosmetic products is a growing concern, particularly in nail and hair salons, where employees and clients are frequently exposed to airborne chemicals. Many beauty products emit harmful pollutants, including volatile organic compounds (VOCs) such as toluene, esters, formaldehyde, and ketones [1]. Airborne particulate matter smaller than 2.5 µm that can easily enter airways is also a major contributor to air pollution within salon settings [1]. These chemicals have been linked to hormone disruption, respiratory issues such as asthma, nervous system disorders, allergies, skin problems, miscarriages, and even cancer [2-5]. Additionally, salon workers are more likely to have low-birth-weight babies and infants born with cleft palates, especially when repeatedly exposed to hairspray and permanent-wave solutions [3]. Proper ventilation and air purification are essential in reducing health risks [6]. The goals of this study were to test how common nail and hair care products affect air quality by measuring air quality index (AQI), total volatile organic compounds (TVOCs), formaldehyde (HCHO), and fine particulate matter (PM₂.₅) levels and to determine the relative effectiveness of air purification and exhaust ventilation methods in restoring air quality.

## Materials and methods

This project received institutional approval and was carried out according to institutional research and safety guidelines. The experiments were conducted in a dedicated, enclosed 270 ft³ room within a building structure used exclusively for testing purposes. The controlled environment approximated the enclosed conditions of a salon workspace while ensuring consistent air quality measurements. The room was equipped with an exhaust fan. A digital air quality monitor measured AQI, TVOC, HCHO, and PM₂.₅ levels. All tested products were approved for household use and were commercially available to the general public. The tested products included a traditional film-forming nail polish, nail polish remover consisting of 100% acetone, aerosol hairspray, and permanent hair dye consisting of a colorant and a developer. The air purification devices used were an exhaust fan operating at 80 cubic feet per minute and an air purifier containing HEPA and activated carbon filters operating at 239 cubic feet per minute. Baseline air quality was measured before testing. For each trial, 1 milliliter of each liquid product (nail polish, remover, hair dye) was applied onto a wax paper. For hairspray, three one-second sprays were released and air-quality parameters were recorded. All four products were then released simultaneously to simulate salon conditions. Each trial was repeated six times, returning the room to baseline between tests. For purification comparison, three configurations were evaluated after all products were simultaneously exposed to air: no purification, exhaust fan activated, and air purifier activated. Air-quality measurements were recorded each minute over a 15-minute period for each configuration, and each test was repeated three times.

Elevations of air quality indicators were expressed as mean ± standard deviation (SD). Statistical difference comparing each product to the clean room (control) was evaluated with Student's t-test. A p-value of < 0.05 was considered statistically significant, and p < 0.001 was highly significant. The results were presented in the form of graphs. Safe limits were indicated on the graphs as 50, 12 µg/m³, 0.3mg/m³, and 0.08ppm for AQI, PM₂.₅, TVOC, and HCHO, respectively. GraphPad (GraphPad Software, San Diego, CA) and Excel were used for the statistical analyses.

## Results

**Figure 1:**
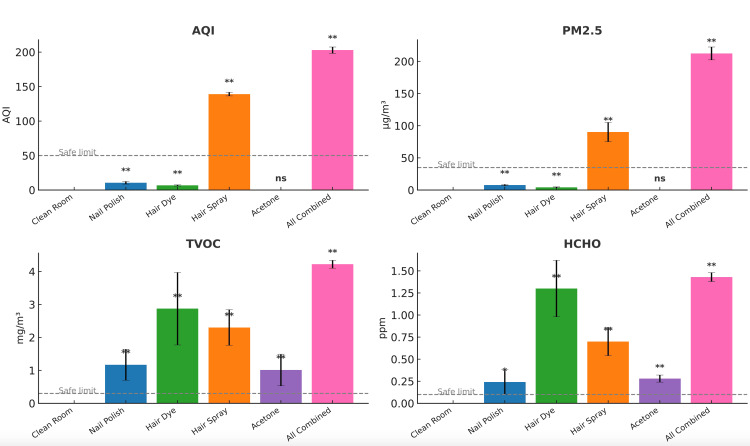
Air quality indicator levels following exposure. AQI, PM₂.₅, TVOC, and HCHO of the clean room and following beauty product exposure. Data presented as mean ± SD (N = 6), *p < 0.05 significant, **p < 0.001 highly significant. TVOC: Total volatile organic compound; AQI: air quality index

**Figure 2:**
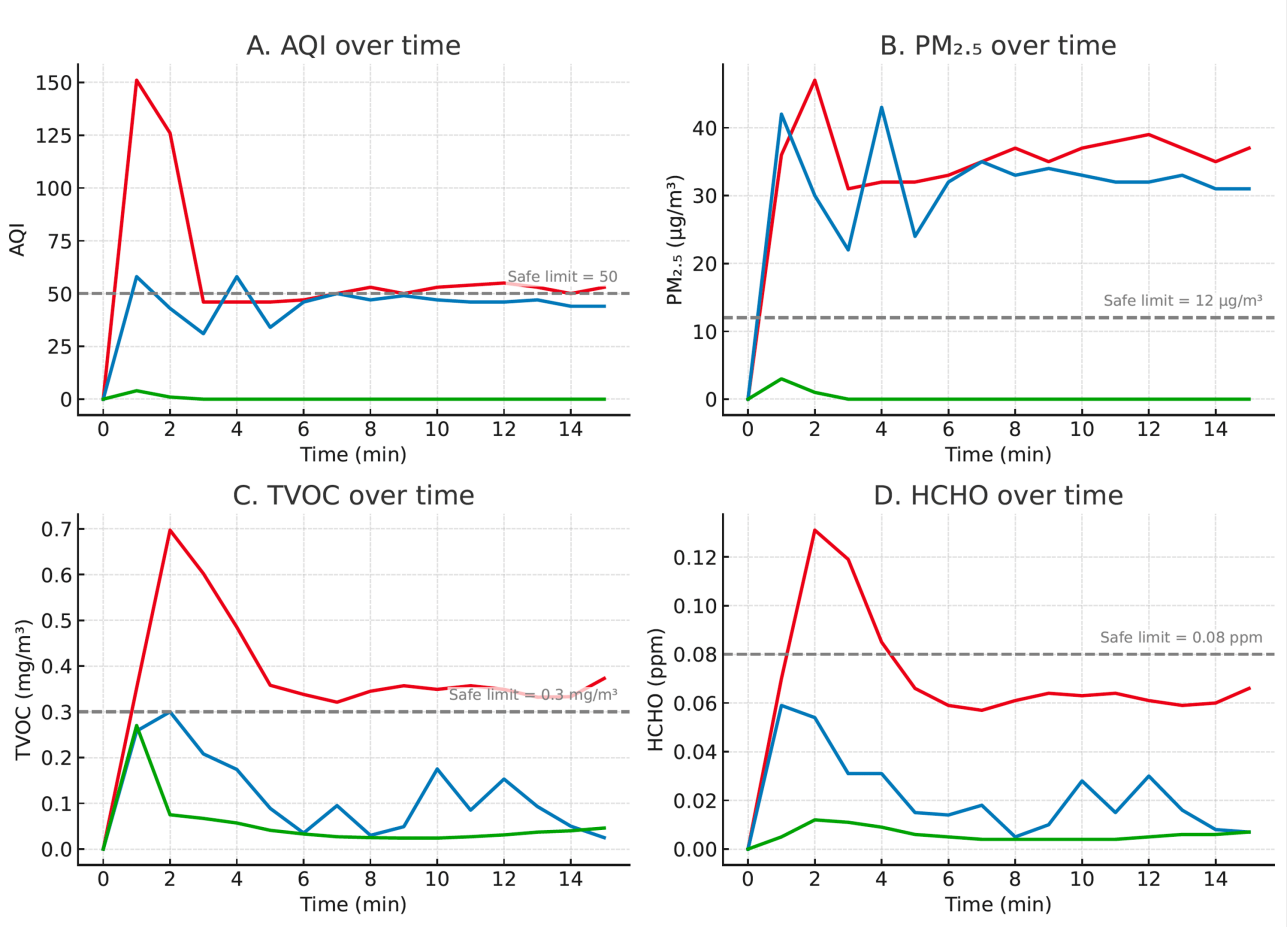
Changes in air quality indicator levels over time. The effects of no purification (red line), exhaust fan ventilation (blue line), and dual carbon-charcoal filter purification (green line) on the levels of air quality indicators after simultaneous exposure to all beauty products. TVOC: Total volatile organic compound; AQI: air quality index

Baseline measurements of the clean control room showed that AQI, TVOC, PM₂.₅, and HCHO levels were zero or near zero prior to product release. Exposure of each product to air resulted in significant elevation of TVOC and HCHO levels above the safe limits. Hairspray release to the air significantly raised the PM₂.₅ level and AQI value above the safe limit. Combined exposure of the tested products resulted in the highest increases of air quality indicator values (Figure 1). Following exposure of all products to air, AQI, TVOC, and PM₂.₅ levels remained elevated for at least 15 minutes. The exhaust fan prevented the elevation of TVOC and HCHO above the safe limit, but not PM₂.₅ levels. The HEPA + activated carbon air purifier was the most effective at attenuating the increase in air pollutants and at restoring the air quality to baseline (Figure 2).

## Discussion

This study demonstrates that routine use of common nail and hair products substantially worsens indoor air quality by elevating AQI, PM₂.₅, TVOCs, and formaldehyde. Among the tested agents, hairspray produced the highest pollutant concentrations, consistent with aerosolized propellants and solvent-based ingredients known to contribute to elevated VOC emissions. Combined use of all products amplified these effects, simulating real salon conditions where multiple products are applied concurrently.

These findings align with prior reports showing that beauty salon environments frequently exceed recommended indoor air pollutant limits [1-3]. Elevated VOC and PM₂.₅ exposure has been associated with irritation and damage of the respiratory system, eyes, liver, and kidneys, as well as with cancer and pregnancy-related complications [7]. Chronic inhalation of compounds such as toluene, acetone, and formaldehyde may result in headaches, mucosal irritation, and reproductive health risks [3-5]. 

The results highlight the superior performance of HEPA and activated carbon air purifiers in rapidly reducing airborne pollutants compared with exhaust ventilation alone. These findings are consistent with prior studies demonstrating that combined particulate and gas-phase filtration markedly lowers PM₂.₅ and VOC concentrations in occupational settings [8,9]. HEPA filters efficiently capture fine particulate matter, while activated carbon adsorbs volatile organic compounds through surface binding. In compact rooms with continuous product use, a purifier provides the most reliable path to quickly restoring air quality to safe levels. The limited performance of the exhaust fan, despite continuous operation, suggests that typical salon ventilation systems may be insufficient for control of aerosolized particulate matter. Therefore, incorporating portable purifiers with dual filtration should be prioritized in beauty service environments.

As observed in this study, AQI increased only when PM₂.₅ levels rose but not with elevated TVOC or HCHO concentrations. AQI values are primarily based on particulate matter, ozone, nitrogen dioxide, and sulfur dioxide concentrations, and not on organic compounds [1]. Therefore, relying solely on AQI measurements is insufficient for assessing air quality in salon environments, where chemical emissions play a major role in exposure risk. The findings in this study underscore the need for effective air quality monitoring and management in beauty salons. Regulatory bodies such as OSHA and the WHO have called for greater emphasis on indoor air quality monitoring and inclusion of air filtration in occupational safety plans [10]. However, there are currently no enforceable federal regulations requiring routine monitoring of AQI, TVOC, HCHO, or PM2.5 in salon environments in the United States. Educating salon staff about proper air purification, product selection, and exposure limits is an essential step toward reducing health risks and improving equity among workers in cosmetology and beauty services. Another proposed solution was to replace the commonly used beauty products with low-VOC or non-toxic alternatives [11]. However, their higher cost has limited their use.

This study had several limitations. The experiments were conducted in a single enclosed room under controlled conditions, which may not fully represent real-world salon dynamics involving larger spaces, open doors, variable temperatures, and human movement. Only one model of air purifier and exhaust fan was tested; therefore, results may not generalize to devices with different airflow capacities or filtration technologies. We did not evaluate long-term or repeated exposure cycles. We also did not speciate VOCs. Future studies should include larger room volumes, variable airflow rates, different purification device types, and testing within actual beauty salons to better reflect real-world conditions.

## Conclusions

Common nail and hair care products significantly worsen indoor air quality by increasing volatile organic compounds, particulate matter, and formaldehyde concentrations. This study demonstrates that HEPA and activated carbon air purifiers effectively remove these pollutants within minutes, outperforming conventional exhaust ventilation. Routine use of filtration-based purification can reduce acute exposure peaks and likely lower cumulative risk for workers and clients. These findings have direct implications for workplace health in the beauty industry.
